# Measurement of Mechanical Properties of VO_2_ Films by Nanoindentation

**DOI:** 10.3390/nano13061042

**Published:** 2023-03-14

**Authors:** Yuemin Wang, Xingang Li, Jiarui Lu, Yao Li, Xiangqiao Yan, Shuliang Dou, Lei Wang

**Affiliations:** 1 Shenzhen Key Laboratory of Polymer Science and Technology, College of Materials Science and Engineering, Shenzhen University, Shenzhen 518060, China; 2 College of Physics and Optoelectronic Engineering, Shenzhen University, Shenzhen 518060, China; 3 Jiangxi Construction Engineering (Group) Corporation Limited, Nanchang 330029, China; 4 School of Engineering, Hong Kong University of Science and Technology, Hong Kong 999077, China; 5 Center for Composite Materials and Structure, Science and Technology on Advanced Composites in Special Environment Laboratory, Harbin Institute of Technology, Harbin 150080, China

**Keywords:** VO_2_ thin films, nanoindentation, intrinsic mechanical behavior, dimensional analysis, finite element model

## Abstract

The present work reported the intrinsic mechanical behavior of vanadium dioxide (VO_2_) thin film deposited on a SiO_2_ substrate using a combination of nanoindentation tests and a theoretical model. The effect of phase transition on mechanical parameters was studied by adjusting the test temperature. A new model that can simultaneously extract the elastic modulus and hardness was derived by introducing a dimensional analysis. The results showed that the thin film exhibits a hardness of 9.43 GPa and a Young’s modulus of about 138.5 GPa at room temperature, compared with the values of 5.71 GPa and 126.9 GPa at a high temperature, respectively. It can be seen that the intrinsic mechanical parameters decrease to a certain extent after a phase transition. Finally, the numerical simulation results were consistent with those of the experiments, which verified the effectiveness of the new method. In addition, this study also provided useful guidance for mechanical tests on other ultra-thin films.

## 1. Introduction

Vanadium dioxide (VO_2_) undergoes a first-order metal-insulator transition with a drastic change in physical properties at around 68 °C [1,2]. The phase transition is always accompanied by a structural transition from the monoclinic (M) phase to the rutile (R) phase, which has potential applications in infrared switches [3], smart thermal control devices [4] and smart windows [5]. In previous studies, researchers have paid more attention to the optimization of thermal, optical and electrical properties after phase transition [6], while mechanical properties have been almost ignored [7]. In fact, phase transition will cause the lattice to expand by 1% along the c_R_-direction, and to shrink by 0.6% and 0.1% along the a_R_-axis and b_R_-axis in the meanwhile [8,9]. Moreover, lattice structure often has a certain relationship with the mechanical behaviors of materials [10]. Therefore, phase transition may affect the mechanical properties, but so far, the basic mechanical parameters of VO_2_ such as elastic moduli and hardness have not been well determined.

VO_2_ are often used in the form of a thin film/substrate, which makes the traditional mechanical testing methods no longer applicable. Nanoindentation has been widely used for characterizing the mechanical properties of micro/nano-scale materials because it is high-resolution and nondestructive [11]. Nanoindentation can be used to research the dependence on the size effect, Hall-Petch effect and strain rate dependence of micro-nano-scale thin films [12,13], and it can even be used in the research on viscoelastic materials [14], but the measurement of basic mechanical parameters, such as of elastic moduli and hardness, is widely used. Generally speaking, the measurement reflects the composite response of the thin film and substrate. In order to avoid the influence of substrate effect, as a rule of thumb, the penetration depth should be less than one tenth of the film thickness. However, for ultra-thin films, when the indentation depth is too small, it is difficult to obtain meaningful results due to the accuracy of the instrument and the passivation effect [15]. Therefore, the issue of how to extract the intrinsic mechanical properties is a key point. Jönsson et al. [16] proposed the relationship between composite hardness and intrinsic hardness according to the weight coefficient of the residual area. Sargent et al. [17] studied a volume mixing model based on the plastic deformation theory. Korsunsky et al. [18] proposed a new theoretical model from the perspective of indentation work. Doerner and Nix [19] suggested that the effective composite elastic modulus in a layered solid can be described as a function of the plane strain elastic modulus. Takeshi Sawa et al. [20] established a method to estimate the intrinsic elastic modulus of film as thin as 10 nm. However, these methods involve too many parameters, resulting in more complex calculations, and sometimes need to be used in conjunction with finite element simulation, which is not conducive to applications.

In this paper, VO_2_ thin films deposited on a SiO_2_ substrate were prepared by magnetron sputtering. Nanoindentation experiments were carried out at room temperature and a high temperature. By combined dimensional analysis and the work distribution of nanoindentation, a new calculation method for extracting intrinsic elastic moduli and hardness was deduced.

## 2. Materials and Methods

VO_2_ thin films were deposited by the magnetron sputtering of a metal vanadium target (99.99%, Φ76.2 mm) on SiO_2_ substrates. In the experiment, the working pressure was set to 0.9 Pa, with a pure argon and oxygen ratio of 80/1.6. The magnetron power was set to 200 W. Additionally, the corresponding power density was 4.38 W/cm^2^. After that, the sample had been thermal-treated at 400 °C for 2 h in order to obtain the M-phase VO_2_ with high crystallinity and uniformity. The thickness of thin film was controlled by the deposition time (~1000 nm in this study).

The crystal structure was measured by Raman microscopy (XploRA PLUS, HORIBA Scientific, Montpellier, France) with a 532 nm laser. By means of an atomic force microscope (Dimension 3100, BRUKER, Bremen, Germany) in the tapping mode, the surface morphology and roughness of the thin film was investigated. The middle infrared transmission spectrum of 2.5–25 μm was measured by a infrared spectrometer (INVENIO-S, BRUKER, Germany). The nanoindentation test (G200, Keysight, Santa Rosa, CA, USA) was carried out at room temperature and a high temperature (100 °C) with a Berkovich diamond tip. The strain rate during loading was set to 0.05 s^−1^ and the thermal drift rate was 0.1 nm/s. Displacement loading was used in the experiment. In order to ensure accuracy, at least ten tests were conducted to calculate the average value of the mechanical parameters. At the same time, for the sake of simplifying the calculation, this paper does not consider the impact of the pressing position and of the adhesion of the thin film to the substrate [21].

## 3. Results and Discussion

### 3.1. Characterizations of the VO_2_ Film

**Figure 1:**
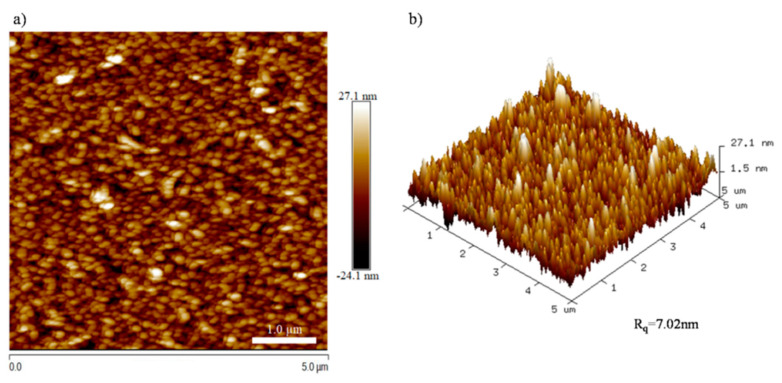
AFM images of VO_2_ thin film (**a**) two−-dimensional image; (**b**) three−-dimensional image.

The surface morphology and surface roughness of the VO_2_ thin film grown on a SiO_2_ substrate was observed by AFM, as shown in Figure 1. It can be seen that the film surface was covered by the uniform grains in the form of a spindle shape, and that the surface roughness was 7.02 nm. The grain sizes were estimated to be 110–240 nm in diameter and about 13 nm in height. This morphology is related to the growth rate of grains being higher than the rate of nucleation, and a similar phenomenon was also observed in other studies [22].

**Figure 2:**
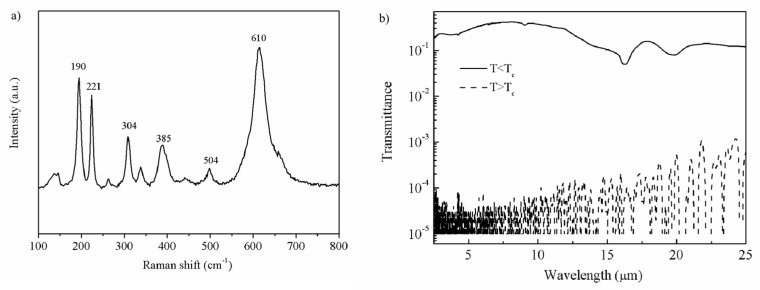
(**a**) Raman spectra of the VO_2_ thin film; (**b**) the transmittance spectra of VO_2_ thin film.

Figure 2a shows the Raman spectroscopy of the VO_2_ thin film. The obvious Raman bands can be found at 190, 221, 304, 385, 504, and 610 cm^−1^, respectively. All Raman bands corresponded to the mode of the VO_2_(M). The symmetry assignment, peak positions and spectral features are similar to those found in other studies [23,24]. Figure 2b shows the optical transmittance spectra over 2.5–25 μm for the VO_2_ thin film in both semi-conductive and metallic states. As expected, the sample showed a higher transmittance in the cold state (T < T_c_) than those in the hot state (T > T_c_). In the mid-infrared region, the transmittance decreased considerably from 42% to 0.1%. In summary, well-crystallized VO_2_(M) thin film was successfully fabricated, as evidenced by the Raman spectra and the transmittance spectra.

### 3.2. The New Model for Extracting Intrinsic Mechanical Parameters

In order to extract the intrinsic elastic modulus and hardness, this paper introduces the dimensional analysis in the analysis of the total work-of-indentation. Assuming that both the film and substrate are ideal elastic-plastic materials, when the indenter is pressed into the sample, the load is a function of the mechanical parameters of the film/substrate system and the geometric parameters of the indenter [25,26], which can be expressed as:

(1) $$P=f_{l}(E_{f},\upsilon_{f},\sigma_{f}{}_{y},E_{s},\upsilon_{s},\sigma_{s}{}_{y},h)$$

where the parameters for the thin film are denoted as $E_{f}$, $\upsilon_{f}$, and $\sigma_{fy}$, while those for the substrate are represented by $E_{s}$, $\upsilon_{s}$, $\sigma_{sy}$, and h as the displacement.

The length, mass and time are selected as the basic units, $E_{com}$ and h are taken as the basic physical quantities, and the other dimensionless quantities are generated by normalizing the unknown parameters by known quantities. By the Buckingham П theorem, Equation (1) can be written as:

(2) $$P=E_{com}h^{2}\prod_{l}(\sigma_{fy}/E_{com},E_{f}/E_{com},\sigma_{sy}/E_{com},E_{s}/E_{com})$$

where $E_{com}$ is the composite elastic modulus, $\prod_{l}=P/E_{com}h^{2}$ is a dimensionless function of $\sigma_{fy}/E_{com},E_{f}/E_{com},\sigma_{sy}/E_{com},E_{s}/E_{com}$.

The total work-of-indentation, $w_{tot}$, to the maximum depth can be calculated by the area under load curve:

(3) $$w_{tot}=\int_{0}^{h}Pdx=\frac{E_{com}h^{3}}{3}\prod_{l}(\sigma_{fy}/E_{com},E_{f}/E_{com},\sigma_{sy}/E_{com},E_{s}/E_{com})$$

In an indentation experiment with a conical indenter, the highest applied load is almost invariably found to relate to the maximum penetration depth by [19]:

(4) $$P=\frac{H_{com}h^{2}}{\kappa }$$

where κ is a parameter describing the indenter geometry, and $H_{com}$ is the composite elastic modulus.

Therefore, Equation (3) can also be written as:

(5) $$w_{tot}=\int_{0}^{h}Pdx=\frac{H_{com}h^{3}}{3}$$

Combining Equations (3) and (5), the relationship between the composite elastic modulus and hardness is as follows:

(6) $$H_{com}=E_{com}\prod_{l}(\sigma_{fy}/E_{com},E_{f}/E_{com},\sigma_{sy}/E_{com},E_{s}/E_{com})$$

Generally speaking, the total work is composed of plastic work and elastic work, but in this paper, the total energy expenditure will now be composed of two parts: the work of the substrate ($W_{s}$) and the energy in the thin film ($W_{f}$):

(7) $$W_{tot}=W_{f}+W_{s}$$

According to Korsunsky’s [13] research,

(8) $$W_{f}=\frac{\lambda H_{f}t^{2}h}{3\kappa }$$

where λ is largely proportional to the coating thickness for plastically deforming coatings, $H_{f}$ is the hardness of thin film, amd t is the thickness of thin film.

Substituting it to Equations (6) and (7), the composite mechanical parameters are obtained in the form of:

(9) $$H_{com}=H_{s}+H_{f}\lambda /{\left(h/t\right)}^{2}$$

(10) $$E_{com}=E_{s}+E_{f}\lambda /{\left(h/t\right)}^{2}$$

where $H_{s}$ is hardness of substrate, and $E_{s}$ is the elastic modulus of substrate.

On the basis of Takeshi Sawa’s model [20]:

(11) $$\frac{1}{E_{com}^{*}}=\frac{1}{E_{s}^{*}}(1-e^{-\gamma (h/t)})+\frac{1}{E_{f}^{*}}e^{-\gamma (h/t)}$$

where γ=0.9 is a fitting constant, and $E^{*}=E/(1-\upsilon^{2})$ is the reduced elastic.

By simultaneously solving Equations (10) and (11), the key parameter λ can be calculated, and then the intrinsic hardness and elastic modulus can be obtained at the same time.

### 3.3. The Nanoindentation Test

**Figure 3:**
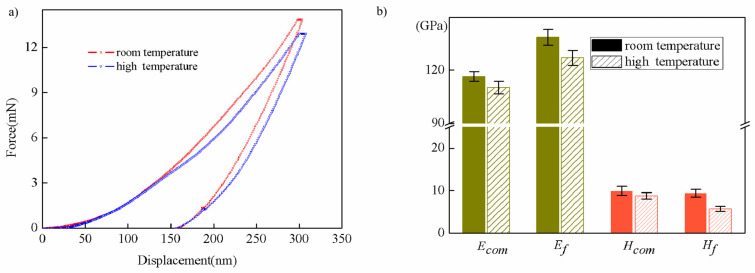
(**a**) The experimental load–displacement curves; (**b**) the composite and intrinsic mechanical parameters.

In order to study the influence of phase transition on mechanical behaviors, room temperature and a high temperature (100 °C) were selected for nanoindentation. The load–displacement curves obtained from the tests are shown in Figure 3a, and the results can be calculated directly by the Oliver–Pharr method [27,28] and the new approach deduced in Section 3.2, as shown in Table 1. The maximum indentation displacement is 300 nm, which is greater than one tenth of the film thickness, so the results are composite mechanical parameters because of the substrate effect.

It can be seen that the intrinsic elastic modulus increases by ~18% compared with the composite elastic modulus at room temperature and by ~15% at a high temperature; in contrast, at room temperature, the intrinsic hardness is reduced by ~5% compared with the composite hardness, but at a high temperature, it is seriously reduced by ~35%. This shows that when the maximum penetration depth is within 1/10, the error caused by the substrate effect cannot be ignored, and the calculation of the intrinsic mechanical properties should be carried out. At the same time, it also can be seen in Figure 3b that the intrinsic elastic modulus and hardness decrease to a certain extent at a high temperature, which may be caused by the change in the crystal structure after the phase transition [10]. This is a reminder that in actual service, for example, when designing smart windows, packaging needs to be designed with consideration of the changes in mechanical parameters, especially the softening after the phase transition.

### 3.4. FEM Simulation

**Figure 4:**
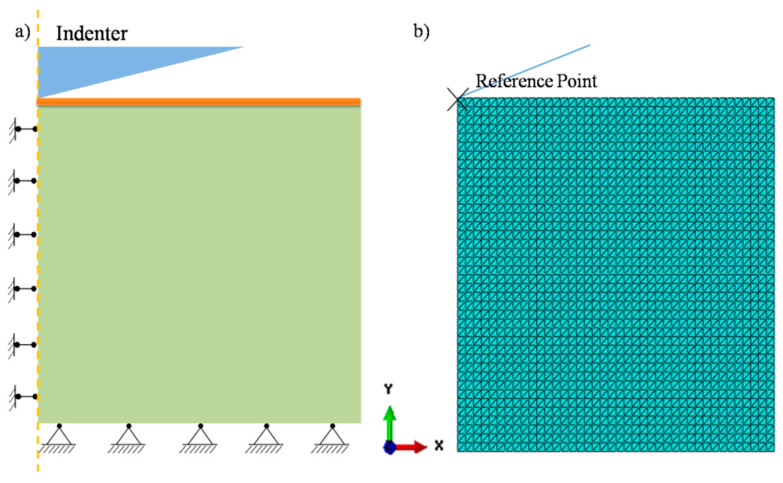
(**a**) The geometric model of film/substrate system; (**b**) the mesh partition of model.

In order to verify the intrinsic mechanical parameters calculated by the new model, FEM was used to simulate a nanoindentation test. All simulation studies were performed by the commercial software Abaqus (version 6.11, Dassault Systems). According to Saint-Venant’s principle, an axisymmetric geometric model with a size of 10 × 10 μm was established as shown in Figure 4a, and the thickness of the film was 1000 nm.

A vertical constraint is imposed on the bottom of the model, and an axisymmetric constraint is imposed on the left axis of symmetry. A CAX3 element is selected for the film and substrate materials, and the triangular mesh is used for free division as shown in Figure 4b. Assuming that the indenter is an ideal rigid body and the thin film yield stress is derived from its relationship with hardness, $\sigma_{fy}\approx H/3.2$ [29,30]. Using a displacement-controlled loading mode, the maximum indentation depth is 300 nm, which is consistent with the result of the test. The contact mode is surface-to-surface, with the rigid indenter as the driving surface and the upper of the tested material as the driven surface.

**Figure 5:**
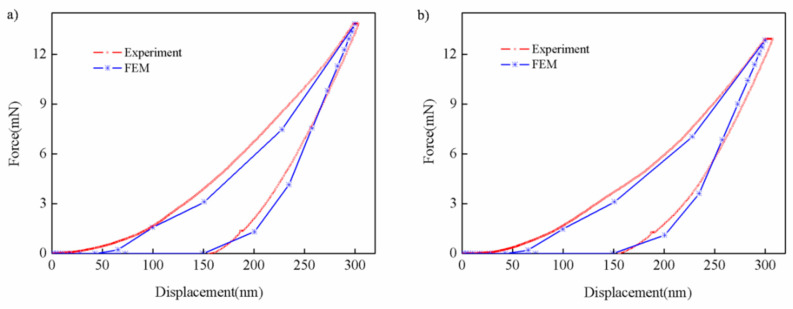
Comparison of experimental curves and finite element predictions for various temperatures: (**a**) room temperature; (**b**) high temperature.

The comparison of the experimental and simulation curves obtained under two temperature conditions is shown in Figure 5. It can be seen that the curves obtained by simulation under the two working conditions were consistent with the load–displacement curves obtained from the tests, and that they verified the rationality of the finite element model. It is worth noting that there is a certain deviation between the experimental curves and the simulation curves in the initial stage of loading (0~70 nm), which is mainly caused by the combined influence of the indenter passivation effect, size effect [31] and other factors. To sum up, the finite element simulation results proved the availability of the new model.

## 4. Conclusions

In conclusion, a thin VO_2_ film was deposited on a SiO_2_ substrate by magnetron sputtering, and nanoindentation experiments were carried out at room temperature and a high temperature. A new model for extracting intrinsic mechanical parameters based on dimensional analysis has been proposed and validated. The results show that the elastic modulus and hardness of the VO_2_ thin film were 138.5 GPa and 9.43 GPa at room temperature, and 126.9 GPa and 5.71 GPa at high temperature, respectively. Both of them reduced to a certain extent, which was caused by the phase transition. FEM simulation was carried out to verify the feasibility of the new method, which also provides a new idea for the mechanical testing of ultra-thin films.

## Figures and Tables

**Table 1:** The composite and intrinsic mechanical parameters of VO_2_ films.

	$E_{com}$/GPa	$E_{f}$/GPa	$H_{com}$/GPa	$H_{f}$/GPa
room temperature	116.4	138.5	9.96	9.43
high temperature	110.2	126.9	8.78	5.71

## Data Availability

All data presented in this study are available upon request from the corresponding author.
